# Long-term prognosis after endoscopic submucosal dissection for colorectal tumors in patients aged over 80 years

**DOI:** 10.1186/s12876-021-01899-y

**Published:** 2021-08-23

**Authors:** Tomoyuki Nishimura, Shiro Oka, Shinji Tanaka, Yuki Kamigaichi, Hirosato Tamari, Yasutsugu Shimohara, Yuki Okamoto, Katsuaki Inagaki, Kenta Matsumoto, Hidenori Tanaka, Ken Yamashita, Yuki Ninomiya, Yasuhiko Kitadai, Koji Arihiro, Kazuaki Chayama

**Affiliations:** 1 Department of Gastroenterology and Metabolism, Hiroshima University Hospital, Hiroshima 1-2-3, Kasumi, Hiroshima, 734-8551 Japan; 2 Department of Endoscopy, Hiroshima University Hospital, Hiroshima, Japan; 3 Department of the Faculty of Human Culture and Science, Prefectural University of Hiroshima, Hiroshima, Japan; 4 Department of Anatomical Pathology, Hiroshima University Hospital, Hiroshima, Japan

**Keywords:** Colorectal tumor, Elderly, Endoscopic submucosal dissection (ESD), Colorectal cancer, The American Society of Anesthesiologists classification of physical status (ASA-PS) class, Long-term prognosis

## Abstract

**Background:**

In Japan, endoscopic submucosal dissection (ESD) is standardized for large colorectal tumors. However, its validity in the elderly population is unclear. We aimed to evaluate the safety and efficacy of ESD for colorectal tumors in elderly patients aged over 80 years.

**Methods:**

ESD was performed on 178 tumors in 165 consecutive patients aged over 80 years between December 2008 and December 2018. We retrospectively evaluated the clinicopathological characteristics and clinical outcomes of ESD. We also assessed the prognosis of 160 patients followed up for more than 12 months.

**Results:**

The mean patient age was 83.7 ± 3.1 years. The number of patients with comorbidities was 100 (62.5%). Among all patients, 106 (64.2%) were categorized as class 1 or 2 according to the American Society of Anesthesiologists classification of physical status (ASA-PS), and 59 (35.8%) were classified as class 3. The mean procedure time was 97.7 ± 79.3 min. The rate of histological *en bloc* resection was 93.8% (167/178). Delayed bleeding in 11 cases (6.2%) and perforation in 7 cases (3.9%) were treated conservatively. The 5-year survival rate was 89.9%. No deaths from primary disease (mean follow-up time: 35.3 ± 27.5 months) were observed. Overall survival rates were significantly lower in the non-curative resection group that did not undergo additional surgery than in the curative resection group (*P* = 0.0152) and non-curative group that underwent additional surgery (*P* = 0.0259). Overall survival rates were higher for ASA-PS class 1 or 2 patients than class 3 patients (*P* = 0.0105). Metachronous tumors (> 5 mm) developed in 9.4% of patients.

**Conclusions:**

ESD for colorectal tumors in patients aged over 80 years is safe. Colorectal cancer-associated deaths were prevented although comorbidities pose a high risk of poor prognosis.

## Background

The elderly population is growing rapidly worldwide, especially in developed countries, leading to increased life expectancy. Elderly people often have comorbidities that pose difficulties when performing surgeries that require general anesthesia for treatment. In recent years, improvements in healthcare technology have increased life expectancy, especially of the elderly population. The main factor contributing to increased mortality of colorectal cancers in Japan is an increase in the morbidity of colorectal carcinomas in the elderly population [1, 2]. Chronic concomitant diseases are common in elderly patients. Further, given that the general condition of elderly patients is inferior to that of younger patients, appropriate treatment options are necessary.

Endoscopic mucosal resection (EMR) is widely performed for the treatment of colorectal tumors [3, 4]; however, piecemeal resection is generally performed for lesions larger than 20 mm in diameter [4–6]. Endoscopic submucosal dissection (ESD) can be performed for complete *en bloc* resection regardless of the tumor size and accurate histopathological diagnosis. This procedure reduces the recurrence rate and is therefore being increasingly employed in many institutions [7–15]. ESD is less invasive than surgery, but colorectal ESD requires considerable experience owing to technical complexity [6, 16–18]. Several reports on the safety and efficacy of colorectal ESD in elderly patients exist [19, 20]. However, it remains unclear how colorectal ESD affects prognosis. In this study, we evaluated the long-term prognosis of ESD for colorectal tumors in patients aged over 80 years with comorbidities.

## Methods

### Patients

This study included 178 lesions in 165 consecutive patients that were resected by ESD in Hiroshima University Hospital between December 2008 and December 2018. All enrolled patients aged over 80 years underwent ESD at the time. Patients who had undergone colectomy or who presented with inflammatory bowel disease, familial adenomatous polyposis, and Lynch syndrome were excluded.

### Compliance with ethical standards

This study was performed in accordance with the Declaration of Helsinki and its later amendments. All patients were informed of the risks and benefits of ESD, and each patient provided written informed consent for the use of their data for publication. This study was approved by the Institutional Review Board of Hiroshima University Hospital (No. 932, registration date: April 25, 2014).

### Indications of ESD

The indications for ESD were as reported previously [7, 8]: (1) lesions for which application of *en bloc* resection with snare EMR was difficult, such as nongranular laterally spreading tumors (particularly the pseudo-depressed type), lesions exhibiting a V_I_-type pit pattern, carcinoma with shallow submucosal invasion, large depressed type tumors, and large protruding type lesions suspected to be carcinoma; (2) mucosal tumors with submucosal fibrosis; (3) sporadic localized tumors in conditions of chronic inflammation such as ulcerative colitis; and (4) local residual or recurrent early carcinomas after endoscopic resection. Before endoscopic therapy, we examined all lesions primarily with magnifying endoscopy [11, 21–24] and determined the indications for ESD or EMR in accordance with the indications provided in the strategy. We performed ESD for lesions that we had diagnosed as deep and submucosally invasive if the patients requested ESD for palliative local cure owing to the severity of their chronic concomitant diseases or malignant diseases. In this study, we only evaluated patients who could be prepared for colonoscopy with more than 1-L bowel cleansing agent; Niflec^®^ (Ajinomoto Co., Inc. Tokyo, Japan).

### ESD procedures

ESD was performed by four endoscopists (S.T., S.O., Y.N., and H.T.). We predominantly used a DualKnife J (Olympus Medical Systems Co., Ltd, Tokyo, Japan), IT knife nano (Olympus Medical Systems Co., Ltd, Tokyo, Japan), or Flex knife (Olympus Medical Systems Co. Ltd, Tokyo, Japan). Depending on the situation, we also used an SB knife Jr. (Sumitomo Bakelite Co., Ltd, Tokyo, Japan). Carbon dioxide (CO_2_) insufflation was used instead of room air insufflation. ESD procedures were performed with a high-resolution magnifying video endoscope (CF-H260AZI, CF-Q260JI, or PCF-H290TI; Olympus Optical Co., Ltd, Tokyo, Japan) or upper gastrointestinal endoscope (GIF- Q260J; Olympus Optical Co. Ltd, Tokyo, Japan). Undiluted 0.4% sodium hyaluronate (MucoUp^®^; Johnson & Johnson K.K., Tokyo, Japan) was used as the injection solution. After injection of the solution into the submucosal layer, a circumferential incision was made using a single ESD knife. The submucosal layer was then dissected using one or two ESD knives. Visible vessels or arteries in the ulcers were grasped precisely with hemostatic forceps.

### Histologic assessment

The excised specimens were stretched and pinned, fixed in 10% buffered formalin, sliced into 2 mm sections, and assessed microscopically. The depth of submucosal invasion was determined according to the General Rules for Clinical and Pathological Studies on Cancer of the Colon, Rectum, and Anus outlined by the Japanese Society for the Colon and Rectum (JSCCR) [25–27]. Lesions were classified as adenoma (including tubular adenoma, tubulovillous adenoma, and serrated adenoma), Tis carcinoma (carcinoma in situ), T1a carcinoma (adenocarcinoma with shallow submucosal invasion [< 1000 μm]), or T1b carcinoma (adenocarcinoma with deep submucosal invasion [≧ 1000 μm]).

A curative resection was determined using the JSCCR guideline criteria, which involved satisfying all four of the following characteristics: a well/moderately differentiated or papillary carcinoma, no lymphovascular invasion, a submucosal invasion depth < 1000 mm, and grade 1 budding. The inclusion of an additional colectomy with lymph node dissection was considered based on the current guidelines at the time [25–27].

### Variables investigated

The following variables for clinical outcomes of ESD were investigated: complete *en bloc* resection, abandoned cases, median procedure time, and complications (delayed bleeding and perforation). Poor scope operability was defined as situations in which paradoxical movement of the endoscope, poor control with adhesions, and lesion motion with heart beats or breathing occurred, as reported previously [28]. A complete *en bloc* resection was defined as a one-piece resection of the entire lesion, as observed endoscopically, and negative margins were defined through histopathological diagnosis.

We compared the prognosis among three groups; curative resection, non-curative resection with additional surgical resection of lymph nodes, and non-curative resection followed up without additional surgical resection. Curative resection, according to the JSCCR Guidelines for the Treatment of Colorectal Cancer [25–27], was defined by histopathological confirmation of well/moderately differentiated or papillary histologic grade lesion-free deep and lateral margins, no vascular invasion, a submucosal invasion depth of < 1000 μm, and grade 1 budding (low grade). Tumor locations were divided into the right colon, left colon, and rectum. Based on their growth patterns, the growth types of the tumors were classified into either superficial or protruding type [29]. We classified the degree of submucosal fibrosis into three groups (F0, F1, and F2) as described previously [16], which were further subdivided into two groups: F0 and F1 were non or mild, and F2 was severe. We used The American Society of Anesthesiologists classification of physical status (ASA-PS) [30, 31] for categorizing the preoperative status of patients before ESD (ASA-PS class 1; a normal healthy patients, ASA-PS class 2; a patient with a mild systemic disease, ASA-PS class 3; a patient with a severe systemic disease that is not life-threatening, ASA-PS class 4: a case of extreme systemic disorders which have already become an imminent threat to life regardless of the type of treatment). We also compared overall survival rates among each ASA-PS class.

### Surveillance after ESD

Follow-up colonoscopy for recurrence was generally scheduled according to the type of resection (curative vs. non-curative). According to the JSCCR guidelines, in cases of curative resection, follow-up colonoscopy for local recurrence was performed once every 12 months. Cases of non-curative resection, which did not undergo additional surgery, were followed up with abdominal ultrasonography and computed tomography in addition to colonoscopy. However, we occasionally changed the period of surveillance according to the patient's physical condition. Confirmation of recurrence was based on imaging and/or pathological findings. Local recurrence was defined as recurrence at the site of resected colorectal tumors. Distant recurrence was defined as the occurrence of metastasis of colorectal origin associated with the initial tumor.

### Statistical analysis

Quantitative data are presented as mean ± standard deviation or percentage. Differences in categorical variables were analyzed with the chi-square test with Yates correction or Fisher’s exact test. A *P* value < 0.05 was ﻿considered statistically significant. The overall survival, disease-free survival, and disease-specific survival rates were calculated using the Kaplan–Meier method. JMP statistical software version 12.2.0 (SAS Institute, Cary, NC, USA) was used for all statistical analyses.

## Results

### Patients and lesion characteristics

The clinicopathologic characteristics of lesions and patients are presented in Table 1. ASA-PS of patients were class 1, 2, and 3 in all cases. There was no patient in ASA-PS class 4. In total, 178 lesions in 165 consecutive patients who underwent ESD for colorectal tumors were evaluated. The mean age of patients was 83.7 ± 3.1 years. The average lesion size was 35.6 ± 18.8 mm. With regard to tumor location, 81 lesions (45.5%) were located on the right side of the colon, 37 lesions (20.8%) were located on the left side of the colon, and 60 lesions (33.7%) were located in the rectum. In total, 69 superficial (38.8%) and 109 protruding growth type (61.2%) lesions were noted. The comorbidity rates were 52.2% for hypertension, 25.5% for cardiac disease, 20.0% for diabetes, 15.2% for cancer of other organs, 6.1% for cerebrovascular disease, 3.6% for chronic kidney disease, 1.8% for liver cirrhosis, and 1.2% for arteriosclerosis obliterans (overlapped). The rate of anticoagulant and/or antiplatelet drug use was 28.7%. Among all patients, 106 (64.2%) were classified as ASA-PS class 1 or 2, and 59 (35.8%) as class 3.

**Table 1:** Clinical characteristics of patients and lesions treated with endoscopic submucosal dissection

Variable	(%)
Number of patients	165
Number of lesions	178
Sex	
Male	94 (57)
Age (years)	83.7 ± 3.1
Tumor size (mm)	35.6 ± 18.8
Location	
Right colon	81 (45)
Left colon	37 (21)
Rectum	60 (34)
Gross type	
Superficial	69 (39)
Protruded	109 (61)
Comorbidities (overlapped)	
Hypertension	86 (52)
Cardiac disease	42 (25)
Diabetes	33 (20)
Advanced-stage cancer of organs	25 (15)
Cerebral vascular disease	10 (6)
Chronic kidney disease	6 (4)
Liver cirrhosis	3 (2)
Arteriosclerosis obliterans	2 (1)
Use of anticoagulants and/or antiplatelet drugs	51 (29)
ASA-PS	
Class 1/2	106 (64)
Class 3	59 (36)

### Outcomes of ESD

All patients underwent ESD with good preparation, without any preparation-related complications. The clinical outcomes of colorectal ESD are presented in Table 2. The mean operative time was 97.7 ± 79.3 min. The rates of *en blo*c resection and histological *en bloc* resection were 95.5% (170/178) and 93.8% (167/178), respectively. The number of cases with good scope operability was 109 (61.2%). The number of cases with severe submucosal fibrosis was 58 (32.6%). Delayed bleeding occurred in 11 cases (6.2%), and perforation occurred in 7 cases (3.9%); all patients were treated conservatively. Three patients had sustained a fall during hospitalization, but no fractures or other minor events were reported. Histologically, 66 lesions (37.1%) were classified as adenoma, 76 (42.7%) as Tis carcinoma, 7 (3.9%) as T1a carcinoma (adenocarcinoma with shallow submucosal invasion [< 1000 μm]), and 29 (16.3%) as T1b carcinoma (adenocarcinoma with deep submucosal invasion [≧ 1000 μm]). The rates of curative resection, non-curative resection with additional surgical resection of lymph nodes, and non-curative resection followed up without additional surgical resection were 83.7%, 9.0%, and 7.3%, respectively. All 15 patients of non-curative resection followed up without additional surgical resection were cases of surgical refusal. Twelve patients refused additional surgery because of old age, and 3 patients refused to receive surgery with permanent colostomy. However, it is not clear whether the reasons for refusing additional surgery are related to the low survival rate, and since they were elderly, most of them died of other concomitant diseases.

**Table 2:** Outcomes of endoscopic submucosal dissection

Variable	(%)
Operation time (min)	97.7 ± 79.3
En bloc resection	170 (96)
Histological en bloc resection	167 (94)
Scope operability	
Good	109 (61)
Poor	69 (39)
Submucosal fibrosis	
Non or mild	120 (67)
Severe	58 (33)
Histopathology	
Adenoma	66 (37)
Tis carcinoma	76 (43)
T1a carcinoma	7 (4)
T1b carcinoma	29 (16)
Progress after endoscopic submucosal dissection	
Curative resection	149 (84)
Consideration for surgery or absolute surgery indication	
Follow-up without surgery	16 (9)
Additional surgery	13 (7)
Complication	
Delayed bleeding	11 (6)
Perforation*	7 (4)

*All patients recovered under conservative therapy

### Prognosis after ESD

We investigated the prognosis of 160 patients (97.0%), excluding 13 duplicate cases and 5 patients with less than 6 months of confirmed survival, out of 178 lesions in 165 consecutive patients (average follow-up period of 35.3 ± 27.5 months).

A total of 25 deaths during prognostic observation were noted (Table 3). Primary cancer death accounted for one patient who required absolute surgery indication due to a positive vertical margin in ESD specimens. The patient refused additional surgery, and recurrence occurred, comprising of lung and liver metastases, within 8 months after ESD. The patient was treated with chemotherapy, which was ineffective, and he died of primary disease 10 months later.

**Table 3:** Cause of death in patients (n = 25)

	(%)
Primary cancer death (recurrence)	
Liver and lung metastasis	1 (4)
Other disease death	
Pneumonia	5 (20)
Heart disease	3 (12)
Oral cancer	2 (8)
Cerebral infraction	2 (8)
Lung cancer	1 (4)
Bladder cancer	1 (4)
Liver cancer	1 (4)
Ovarian cancer	1 (4)
Malignant melanoma	1 (4)
Others	7 (28)

**Fig. 1:**
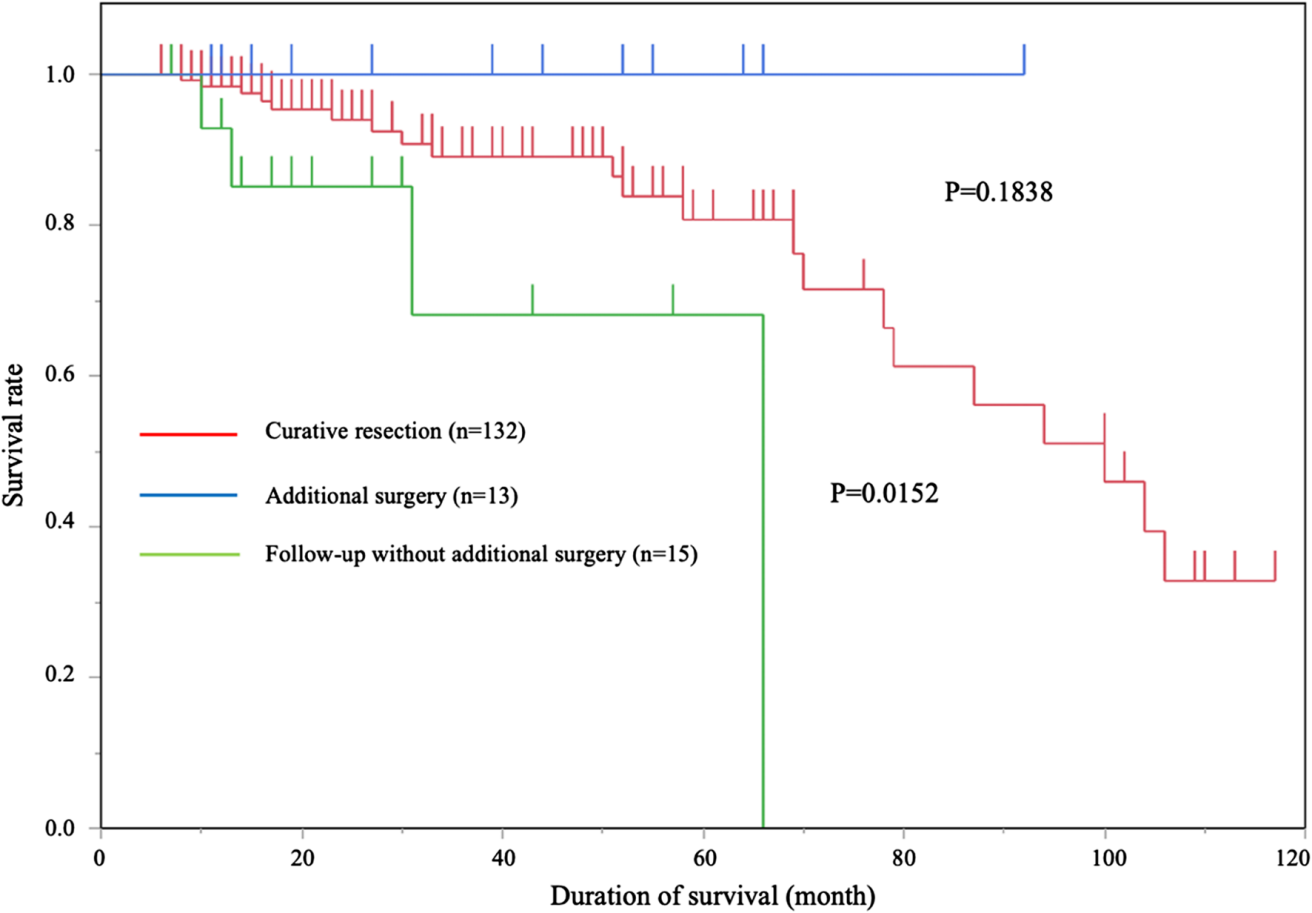
Kaplan–Meier curves for overall survival rates

The overall survival rate for all patients was 94%. No deaths were observed in the non-curative resection with additional surgical resection group during the observation period. The overall survival rates for curative resection, non-curative resection with additional surgical resection of lymph nodes, and non-curative resection followed up without additional surgical resection are presented in Fig. 1. No significant difference was observed between curative resection and non-curative resection with additional surgical resection groups (*P* = 0.1838). A significant difference was observed between curative resection and non-curative resection followed up without additional surgical resection groups (*P* = 0.0152). A significant difference was observed between non-curative resection with additional surgical resection and non-curative resection followed up without additional surgical resection groups (*P* = 0.0259).

**Fig. 2:**
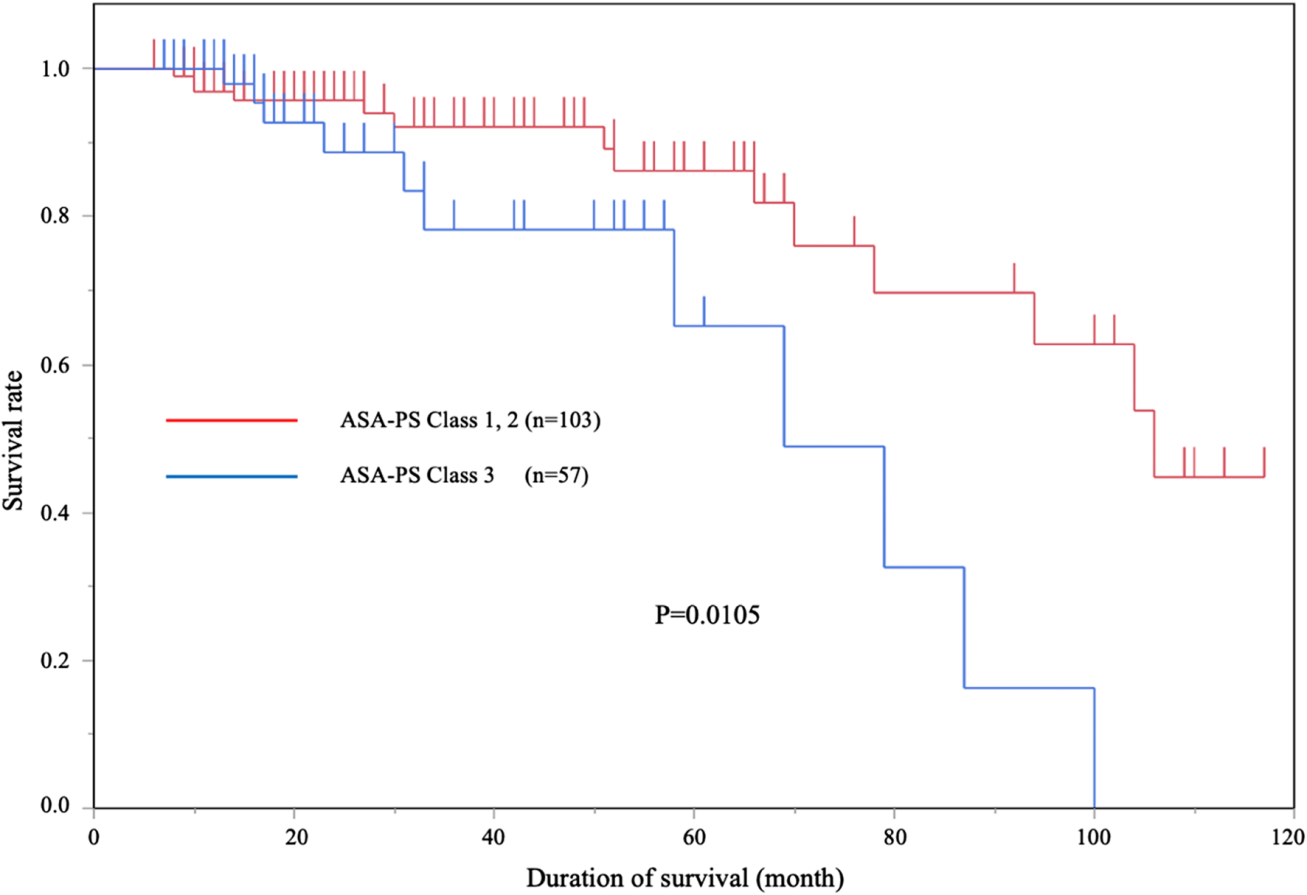
Prognosis of patients according to ASA-PS (n = 160)

**Fig. 3:**
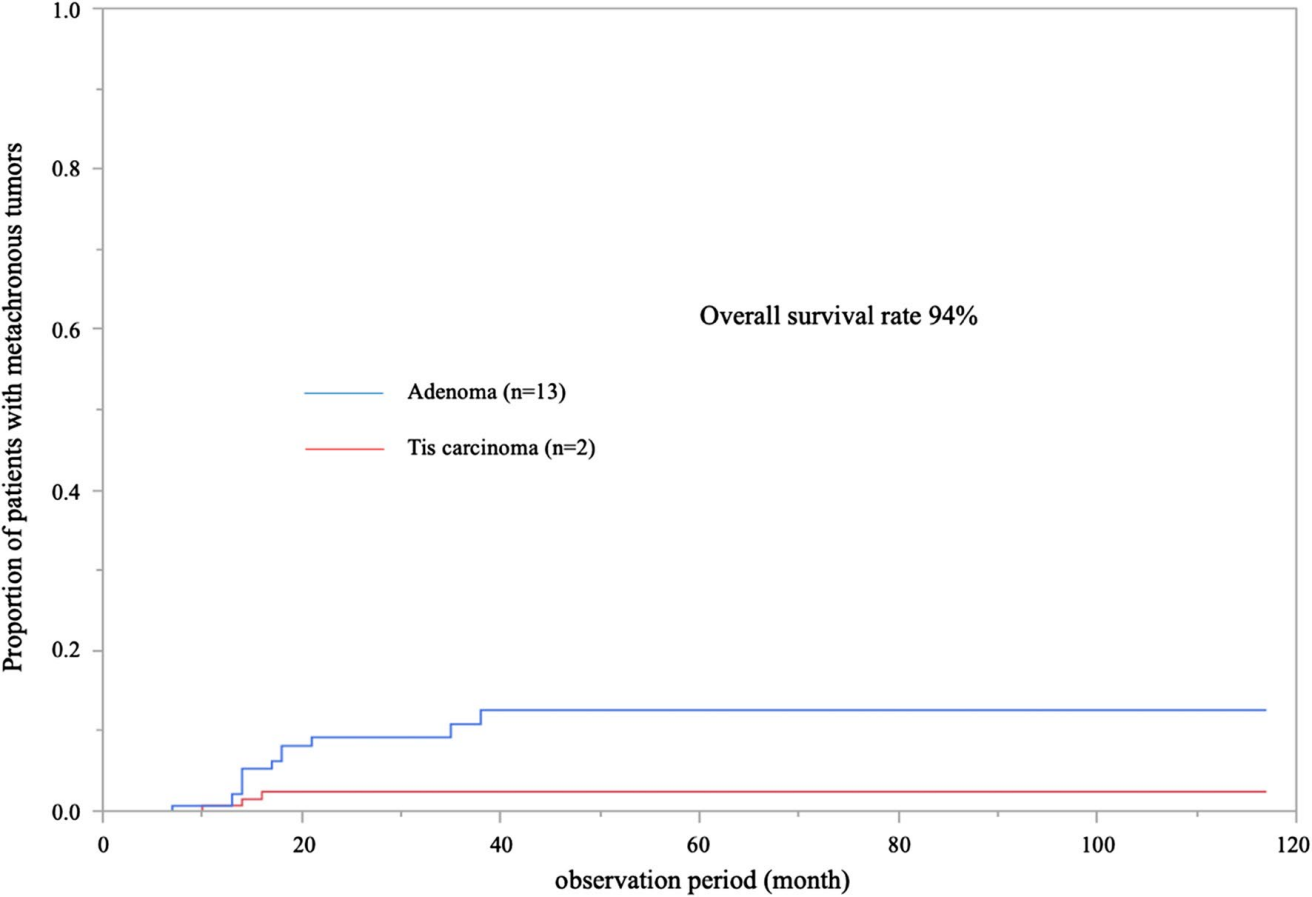
Rates of metachronous tumor after ESD

The overall survival rates according to ASA-PS are depicted in Fig. 2. Among all patients, 103 were classified as ASA-PS class 1 or 2, and 57 were classified as class 3. A significant difference was observed between ASA-PS class 1/2 and 3 (*P* = 0.0105). The rate of metachronous tumors after ESD is presented in Fig. 3. Tumors discovered more than 2 months after ESD were defined as metachronous tumors. Adenoma and Tis carcinoma were observed in 13 and 2 patients, respectively. All lesions were treated by EMR or re-ESD.

## Discussion

This study examined the long-term prognosis of colorectal ESD in the elderly population aged over 80 years. We previously reported on a multicenter study of the clinical outcomes of ESD for colorectal tumors [32]. However, a limited number of reports have focused on elderly patients. Comparing this report to previous reports [33–35], no differences in treatment outcomes were observed between patients of all ages, including the elderly. The 5-year survival rates were lower for elderly patients than for patients of all ages; however, only one patient with primary disease death was identified. These results suggest that ESD is highly effective and safe in elderly patients. We focused on elderly patients over 80 years old. Takahashi et al. compared the outcomes of all patients between an elderly group (≥ 75 years of age) and a non-elderly group (< 75 years of age) [36]. They included patients who underwent additional surgery and those who were followed up without surgery. Our study divided the patients into three groups—curative resection group, additional non-curative resection group, and non-curative resection follow-up group—according to the comorbidities of patients by ASA-PS. In the present study, the overall survival rate was significantly lower for patients who underwent non-curative resection followed up without additional surgical resection than for patients who underwent curative resection and non-curative resection with additional surgical resection. This suggests that patients for whom additional surgery is not possible may have a poorer prognosis than other patient groups. Most of the deaths were due to causes other than colorectal carcinoma. Furthermore, the overall survival rate after ESD was significantly lower for patients classified as ASA-PS class 3 than for patients classified as ASA-PS class 1 or 2. Although ESD can be safely performed in patients with comorbidities, the likelihood of eventual death from other causes is high. We previously reported on the long-term prognosis after ESD in elderly gastric cancer patients [37]. Gastric ESD can be performed safely even in elderly patients [37]. Nevertheless, although it can prevent gastric cancer-related deaths, it carries a high risk of poor prognosis in patients with comorbidities.

Colorectal ESD was performed as an advanced medical treatment without National Health Insurance coverage until March 2012 in Japan. In April 2012, the National Health Insurance Scheme began offering coverage for expenses incurred for undergoing colorectal ESD. Laparoscopy-assisted colectomy is also performed as a minimally invasive surgical procedure for colorectal cancers. The advantages of laparoscopy-assisted colectomy in the elderly have been reported in other studies [38]. However, laparoscopy-assisted colectomy has several disadvantages including the need for general anesthesia and higher invasiveness than that of ESD [39]. Other minimally invasive surgical procedures for rectal lesions are transanal endoscopic microsurgery (TEM) and transanal resection (TAR). However, due to the narrow field of vision, TEM (3–7%) and TAR (23%) are associated with higher local recurrence rates than ESD (0–2%) [40–43]. Although EMR for colorectal tumors is widely performed [3, 4], *en bloc* resection is difficult to perform on tumors larger than 20 mm in diameter, and piecemeal resection is often adopted [4–6]. Several studies have demonstrated that ESD requires a longer procedure time and is associated with a higher perforation rate than that of EMR and piecemeal EMR, but is associated with a lower local recurrence rate (0–2%) than that of piecemeal EMR (7.4–20.1%) [3, 6, 44–48].

We previously reported the lack of carcinoma incidence and high-grade dysplasia after 79 years of age, and relatively low cumulative incidence of the target lesion [49]. This study demonstrated that the rate of metachronous tumors after ESD was only 9.4% (adenoma: 8.1%, Tis carcinoma: 1.3%), and all lesions were treatable by EMR or ESD. As the risk of new lesions in the elderly is low, we believe that performing ESD for treatment-eligible lesions in patients who can be prepared for colonoscopy is useful. In conjunction with our previous report [49], our date shows that follow-up with annual colonoscopy may be unnecessary after ESD for treatment-eligible lesions in patients aged over 80 years.

This study has several limitations. First, this study was a retrospective and single-institution study based on clinical records. The statistical power of this study may be less due to the small sample size. Second, it was not possible to follow all patients who underwent ESD. Third, we did not compare elderly patients to young patients. Therefore, we were unable to compare similar situations in different age groups. Fourth, all patients included in this study were post-ESD patients; patients who were not treated despite existing treatment-eligible lesions were not investigated.

## Conclusion

Our study demonstrated that ESD prevented colorectal cancer-related deaths in patients aged over 80 years, regardless of their comorbidities. Further studies, including randomized control trials and larger sample sizes, are needed to elucidate the safety and effectiveness of colorectal ESD in elderly patients with different comorbidities.

## Data Availability

All data generated or analyzed during this study are included in this published article and its supplementary information files.

## Abbreviations

EMR: Endoscopic mucosal resection
ESD: Endoscopic submucosal dissection
JSCCR: Japanese Society for the Colon and Rectum
ASA-PS: Anesthesiologists classification of physical status
Tis carcinoma: Carcinoma in situ
T1a carcinoma: Adenocarcinoma with shallow submucosal invasion [< 1000 μm]
T1b carcinoma: Adenocarcinoma with deep submucosal invasion [≧ 1000 μm]
TEM: Transanal endoscopic microsurgery
TAR: Transanal resection
